# Improving the Recovery of Phenolic Compounds from Spent Coffee Grounds (SCG) by Environmentally Friendly Extraction Techniques

**DOI:** 10.3390/molecules26030613

**Published:** 2021-01-25

**Authors:** Ilhami Okur, Betul Soyler, Purlen Sezer, Mecit Halil Oztop, Hami Alpas

**Affiliations:** 1 Department of Food Engineering, Middle East Technical University, 06800 Ankara, Turkey; ilhami.okur@metu.edu.tr (I.O.); betulsoyler06@gmail.com (B.S.); purlen.sezer@metu.edu.tr (P.S.); mecit@metu.edu.tr (M.H.O.); 2 Department of Food Engineering, Niğde Ömer Halisdemir University, 51240 Niğde, Turkey

**Keywords:** spent coffee grounds (SCG), phenolic compounds, high hydrostatic pressure-assisted extraction (HHPE), ultrasound-assisted extraction (UAE)

## Abstract

The aim of this study was to investigate and compare the effects of different extraction techniques (high hydrostatic pressure-assisted extraction (HHPE), ultrasound-assisted extraction (UAE), and classical solvent extraction (CSE)) on phenolic compounds from spent coffee grounds (SCG). Different HHPE parameters (300, 400 and 500 MPa at 25 °C for 5, 10 and 15 min) and UAE parameters (40%, 50%, and 60% amplitude at 25 °C for 5, 10 and 15 min) were used. These techniques were compared with CSE (at 50 °C for 30 min) according to total phenolic content (TPC), antioxidant activity (AA), high-performance liquid chromatography (HPLC), scanning electron microscopy (SEM), and infrared (IR) spectroscopy. The results showed that eco-friendly techniques increased the TPC and AA compared to CSE and morphological changes were verified by SEM results. Furthermore, chlorogenic and caffeic acid were also quantified by using HPLC. Chlorogenic acid was found as the main phenolic compound in spent coffee grounds (SCG). The highest chlorogenic acid was detected as 85.0 ± 0.6 mg/kg FW with UAE at 60% amplitude for 15 min. In brief, for the extraction of phenolic compounds from waste SCG eco-friendly techniques such as HHPE and/or UAE were more convenient than CSE.

## 1. Introduction

Food waste is defined as any food industry outputs that are neither used for defining end-products nor for alternative purposes such as recycling [1]. A huge amount of food waste is generated every day and it is assumed that up to one-third of the food produced is wasted around the world. Therefore, food waste valorization is important and it is in the core of many studies [2]. Coffee is not only one of the most famous beverages but also it is the second most commercialized product across the world [3]. Spent coffee ground (SCG) is the main waste of processing roasted coffee powder in hot water or steam [4,5]. In recent years, there has been many studies related to the presence of phytochemicals in SCG, therefore SCG might be a source of valuable waste products because of the presence of mainly phenolic compounds such as caffeic and chlorogenic acids which might be used as natural antioxidants, in different industries such as food, cosmetics and pharmaceuticals [4,5,6,7,8].

In the extraction processes, choosing the appropriate extraction method is important [9]. Conventional extraction techniques such as solvent extraction need a lot of time, solvent and energy [10]. Therefore, the development and the usage of environmentally friendly extraction methods have become popular due to their reduced solvent consumption, decreased energy usage, shorter operation time and higher extraction yield [11,12,13]. Some of these techniques are high hydrostatic pressure-assisted extraction (HHPE) and ultrasound-assisted extraction (UAE). High hydrostatic pressure is a novel technique that is performed between 100 and 900 MPa to destroy microorganisms, vegetative cells and enzymes to extend the shelf life of food products [14]. It is commonly used in the food industry as an alternative method to heat treatment [15,16]. In HHPE, a large differential pressure gradient is obtained between the interior and exterior cell membranes that causes rapid permeation [17]. Also, HHPE can be performed at room temperature (cold extraction) and this prevents the degradation of heat-sensitive compounds [18]. On the other hand, UAE, considered as a green extraction technique, basically involves using sound waves within the frequency range of 20 kHz to 100 MHz [19]. This technique has already been used for biologically active compounds because of the decreased extraction time, low solvent consumption and improved efficiency [20]. During UAE, cavitation occurs which produces bubbles during the period of negative pressure, which are then compressed causing their collapse. Then, solid cell walls are disrupted facilitating the release of bioactive compounds [21].

To the best of our knowledge, the application of HHPE and UAE on the polyphenol extraction from SCG has not been reported simultaneously. Hence, the aim of this study was to: (i) investigate the effects of eco-friendly extraction techniques (such as HHPE and UAE) on the extraction of spent coffee grounds (SCG) (ii) find the effect of extraction parameters of these techniques on the extraction of phenolic compounds from SCG (iii) to compare these techniques with conventional solvent extraction (CSE) by examining on total phenolic content, antioxidant activity, IR spectra, scanning electron microscopy (SEM) images and high-performance liquid chromatography (HPLC).

## 2. Results and Discussion

### 2.1. Total Phenolic Content (TPC)

**Figure 1:**
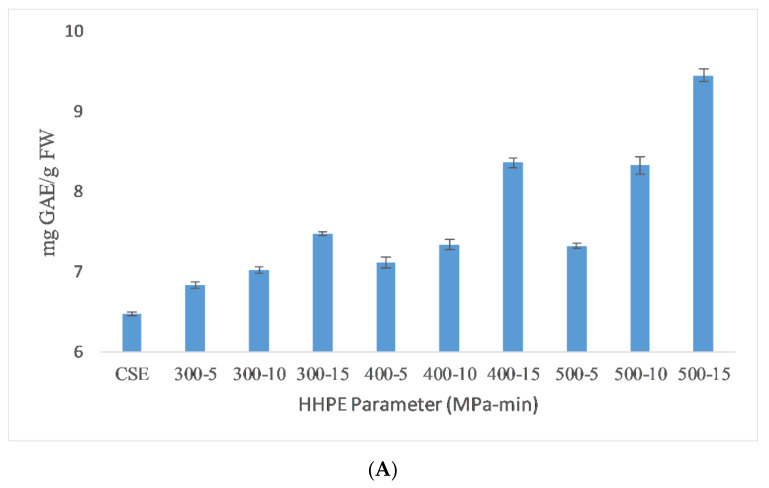

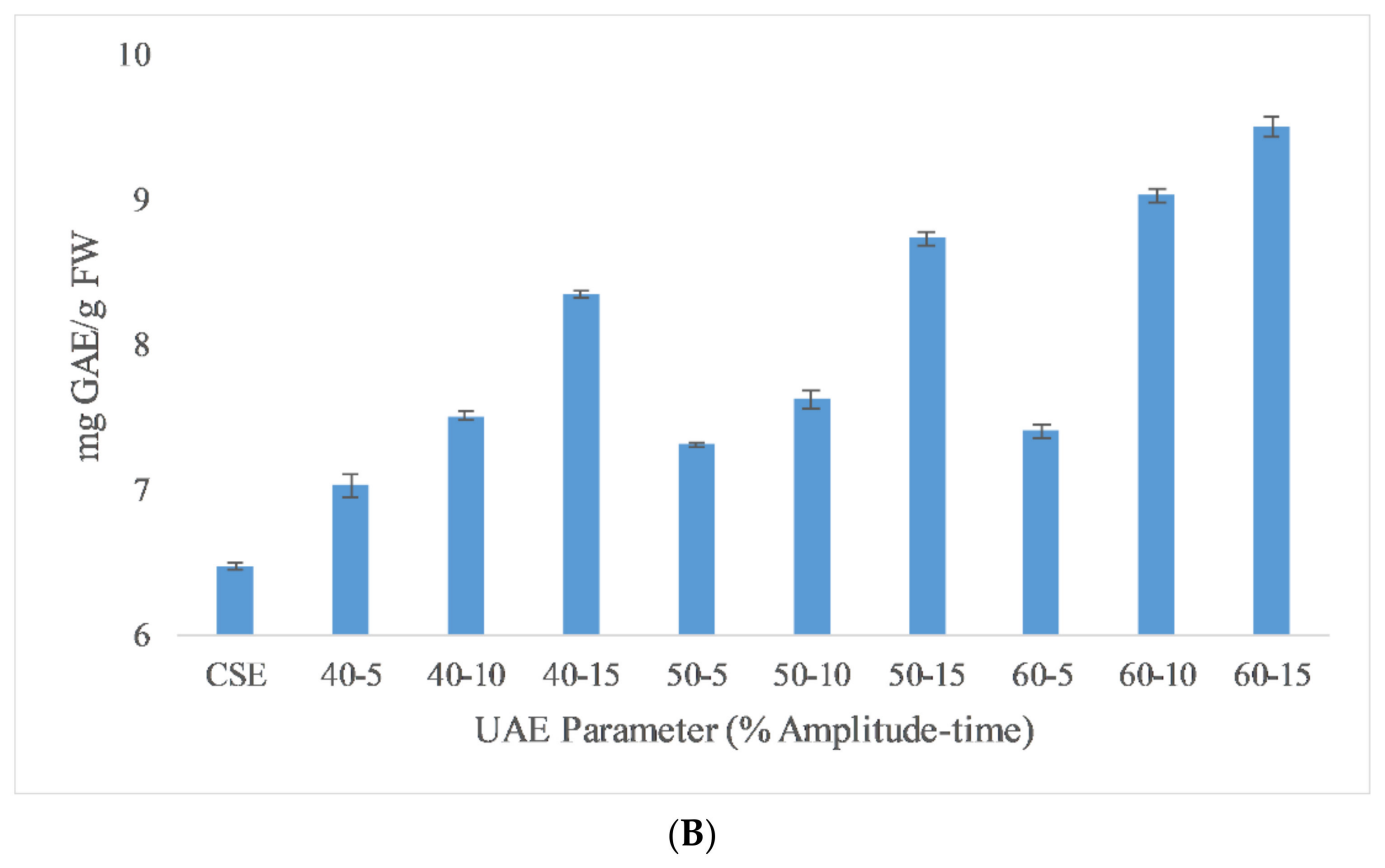
TPC results of different extraction techniques (**A**) HHPE treatment (**B**) UAE.

The total phenolic content (TPC) results of the different extraction techniques are shown in Figure 1. As seen in the figure, HHPE and UAE treatments increased the TPC content compared to CSE. UAE-treated samples at 60% amplitude for 15 min had the highest TPC (9.51 ± 0.06 mg GAE/FW). TPC was increased significantly (*p* ≤ 0.05) with UAE amplitude and time. When the amplitude was increased, a higher amplitude of waves traveling through the liquid media occurred and a large number of bubbles were created that crumbled more violently with enhanced cell disruption and the release of intracellular components [22]. Caballero-Galván et al. [23] also reported that UAE increased the TPC compared to solvent and soxhlet extraction which is parallel to our findings.

HHPE increased TPC significantly (*p* ≤ 0.05) as the pressure and time were increased, affecting the hydrophobic bonds in cellular membranes and resulting in an increase on the mass transfer rate and accordingly leading to an increase in the TPC content [9,24]. Higher pressure levels favor solvent penetration into the cells and more phenolics were extracted. In addition, longer treatment times resulted in an increase in the amount of phenolic substances obtained. Similar results were also reported in literature with Manuka honey (200, 400 and 600 MPa for 5, 10 and 15 min), sour cherry pomace (400 and 500 MPa for 1, 5, and 10 min) and olive pomace (300 and 600 MPa for 5 and 10 min) [9,25,26]. The extraction of phenolic compound from SCG has been widely studied by other techniques. It was reported that TPC value of the low-grade green coffee and spent coffee was between 1.0 and 4.5 mg GAE/g dry weight in a water bath (70 °C) for 10 min [27]. Ballesteros et al. [5] found that the optimum autohydrolysis conditions to extract phenolic compounds from SCG was 40.36 mg GAE/g SCG at 200 °C for 50 min with a liquid/solid ratio of 15 mL/g. The result of this study was much higher than our findings due to extraction temperature and time.

### 2.2. Antioxidant Activity (AA)

#### 2.2.1. DPPH Assay

**Figure 2:**
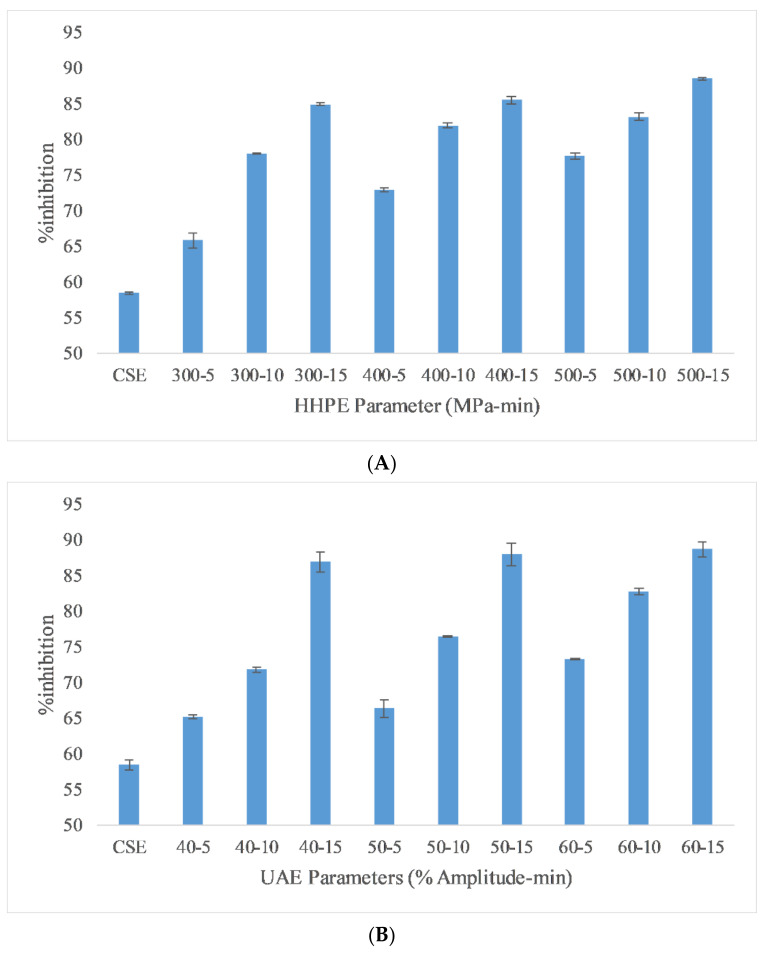
DPPH assay results of (**A**) HHPE treatment (**B**) UAE.

DPPH assay results of different extraction techniques are reported in Figure 2 to depict AA. Like the TPC results, CSE had the lowest DPPH assay activity and HHPE and UAE caused an increase in DPPH assay activity. For HHPE, the antioxidant activity increased statistically significantly (*p* ≤ 0.05) with increasing pressure and time. Furthermore, UAE also increased antioxidant activity statistically significantly with amplitude and time (*p* ≤ 0.05). Generally, the antioxidant activity is proportional to the total phenolic content (TPC) of the extracts [28]. According to Pearson correlation, a strong correlation (0.816) was also found between antioxidant activity and TPC. Caballero-Galván et al. [24] showed that UAE increased the AA of SCG compared to solvent and Soxhlet extraction and this result also agrees with our findings. Okur et al. [26] also reported that HHPE showed higher AA than CSE in sour cherry pomace.

#### 2.2.2. Ferric Reducing Antioxidant Power (FRAP)

**Figure 3:**
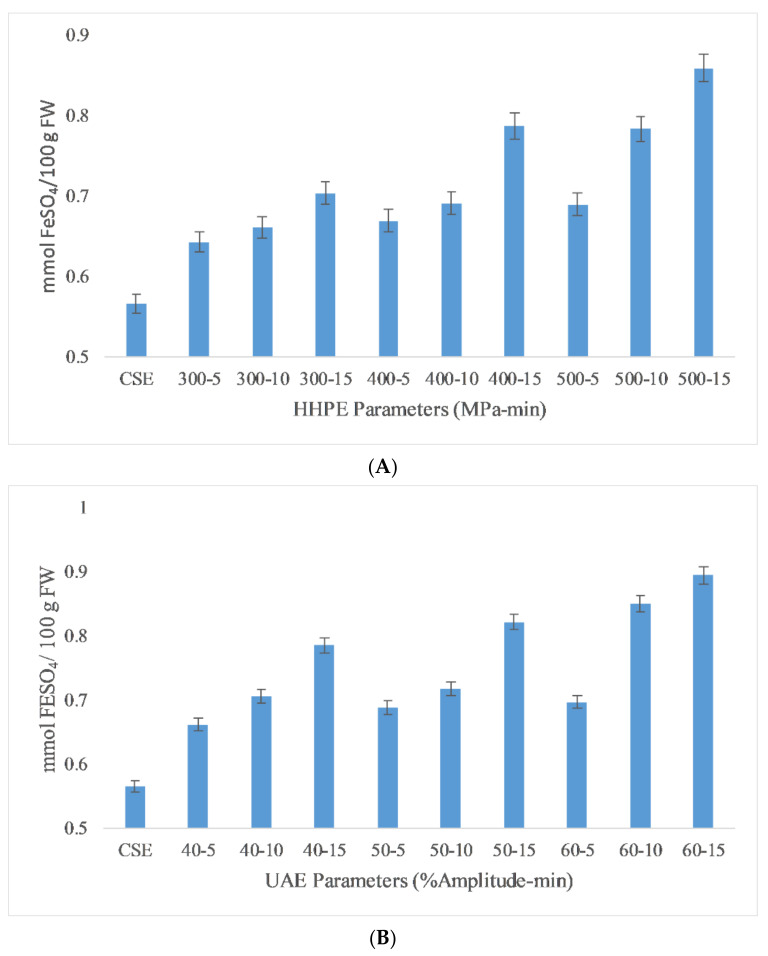
FRAP results of different extraction techniques (**A**) HHPE treatment (**B**) UAE.

Ferric reducing antioxidant power (FRAP) is a useful method to measure the antioxidant activity (AA) of extracts due to its low cost, speed and technical simplicity [29]. The FRAP results of the different extraction techniques are reported in Figure 3. The lowest antioxidant activity was found in CSE at 0.57 ± 0.05 mmol FeSO_4_/100 g FW while the highest antioxidant activity (AA) was found in UAE at 60% amplitude for 15 min as 0.89 ± 0.04 mmol FeSO_4_/100 g FW. According to the Pearson correlation, a strong correlation (0.9) was detected between AA and TPC. Also, eco-friendly techniques gave more antioxidant activity than CSE. In HHPE, the volume of a system tends to reduce whwhen the pressure is increased from atmospheric pressure to operating temperature. During the HHPE process, the extraction solvent enters into cells to interact with the bioactive components. Furthermore, the permeability of pressurized cells increases. Thus, more phenolic compounds permeate out into the solvent when the operating pressure is increased and the more solvent enters the cells [29]. At UAE, the hydrodynamic force causes the disruption of the cell walls. When the amplitude is increased, more extensive cavitation occurs and this improves the release rate of phenolic compounds from the sample into the solvent [30].

### 2.3. High Performance Liquid Chromatography (HPLC)

**Figure 4:**
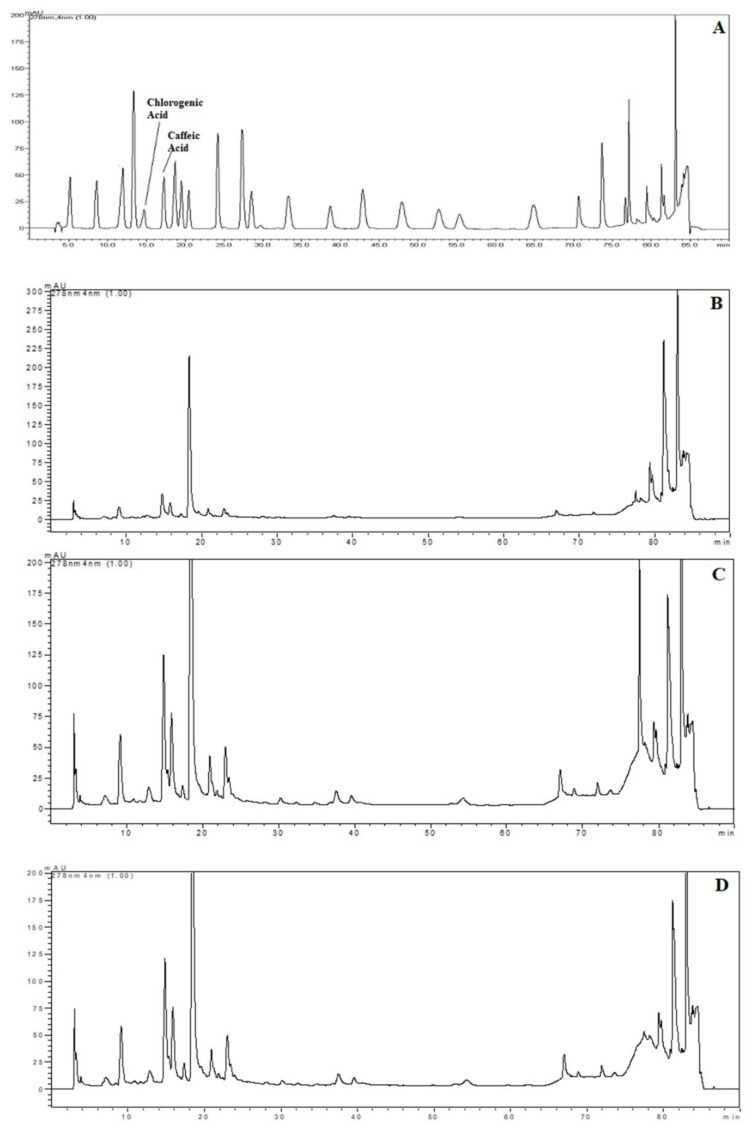
HPLC chromatograms (**A**) Chromatogram of phenolic standards (**B**) Chromatogram of phenolic from SCG extracted by CSE (**C**) Chromatogram of phenolic from SCG extracted by HHP treatment (500 MPa for 15 min) (**D**) Chromatogram of phenolic from SCG extracted by UAE (60% Amplitude for 15 min).

The samples were chosen for HPLC analysis according to the highest TPC and AA results with each extraction technique. For these samples, chlorogenic and caffeic acid content were detected and quantified by using HPLC. Chromatograms of the standards and phenolics from extracts obtained using HHPE, UAE and CSE are shown in Figure 4. The quantities of the phenolic compounds are reported in Table 1. Much more chlorogenic acid than caffeic acid was obtained with all the extraction methods. It was also reported as the major compound in SCG in the literature, [31,32,33]. The highest chlorogenic acid content was found in UAE at 85.0 ± 0.6 mg/kg FW, while the lowest content was found in CSE at 24.0 ± 0.3 mg/kg FW. Like chlorogenic acid content, the highest caffeic acid content was found in UAE (6.1 ± 0.2 mg/kg FW) and the lowest caffeic acid was found in CSE (2.2 ± 0.1 mg/kg FW). Furthermore, it was obvious that the eco-friendly techniques both increased the caffeic and chlorogenic acid contents significantly (*p* ≤ 0.05). UAE had higher caffeic and chlorogenic acid content as compared to HHPE. This result also matched the TPC and antioxidant activity results. At UAE, acoustic cavitation phenomena and the bubbles generated damaged the cell walls. On the other hand, in HHPE, the rapidly increasing pressure caused an acceleration of cell wall breakage and solvent penetration to the cells. In the literature, more phenolic content was observed at UAE than HHPE for sour cherry pomace, olive pomace and tomato peel waste [9,26,34]. Caballero-Galván et al. [24] indicated that UAE increased the chlorogenic and caffeic acid content comparing to solvent extraction and Soxhlet extraction. This result matches our findings.

### 2.4. Infrared (IR) Spectroscopy

**Figure 5:**
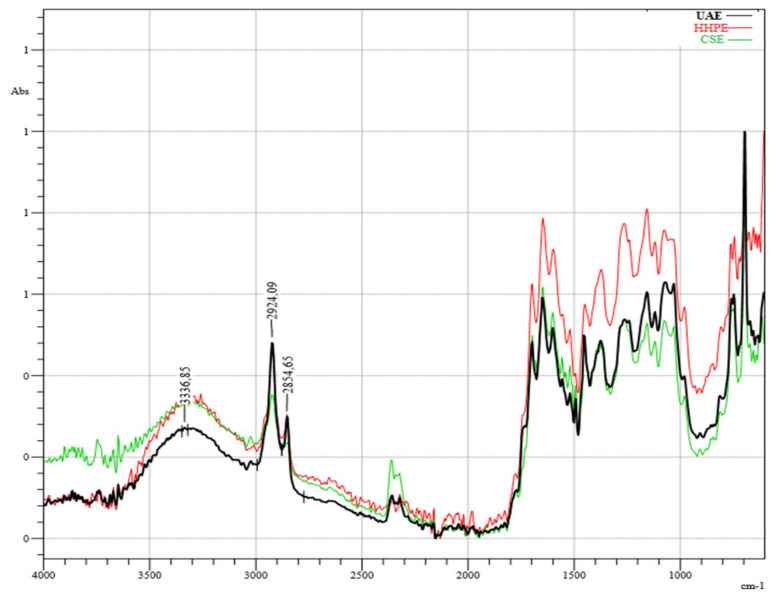
Infrared (IR) Spectrum results. UAE (60% Amplitude for 15 min), HHPE (500 MPa for 15 min), CSE.

The infrared (IR) spectroscopy analysis of SCG extractions using different techniques is shown in Figure 5. According to the results, there was no destruction of the chemical structures of phenolic compounds caused by the environmentally-friendly extraction techniques HHPE and UAE or CSE. The broad band around 3400 cm^−1^ corresponds to OH-stretching with a minor contribution of -NH functional groups [35]. The bands at 1523 cm^−1^ and 1655 cm^−1^ correspond to the C=C vibrations of aromatic rings from lignin moieties and the C=C vibrations of unsaturated lipids and fatty acids, respectively [36]. The sharp peaks at 2925 cm^−1^ and 2855 cm^−1^, showing the presence of methyl and methylene groups, respectively, sre related to the asymmetric and symmetric stretching of C-H bonds in aliphatic chains [37]. In the literature, the presence of caffeine was explained with these peaks [38]. The bands between 1061 cm^−1^ and 1376 cm^−1^ are related to chlorogenic acids formed by quinic acid and *trans*-cinnamic acids; respectively [39]. Moreover, the bands between 900 cm^−1^ and 1400 cm^−1^ show different types of vibrations consisting of the C-H, C-O C, C-N and P-O bonds characteristic of polysaccharides [40]. Although it was difficult to detect the corresponding bands due to either to chlorogenic acids or polysaccharides, HPLC analysis of this study showed that the extracts contained chlorogenic acid.

### 2.5. Morphological Analysis

**Figure 6:**
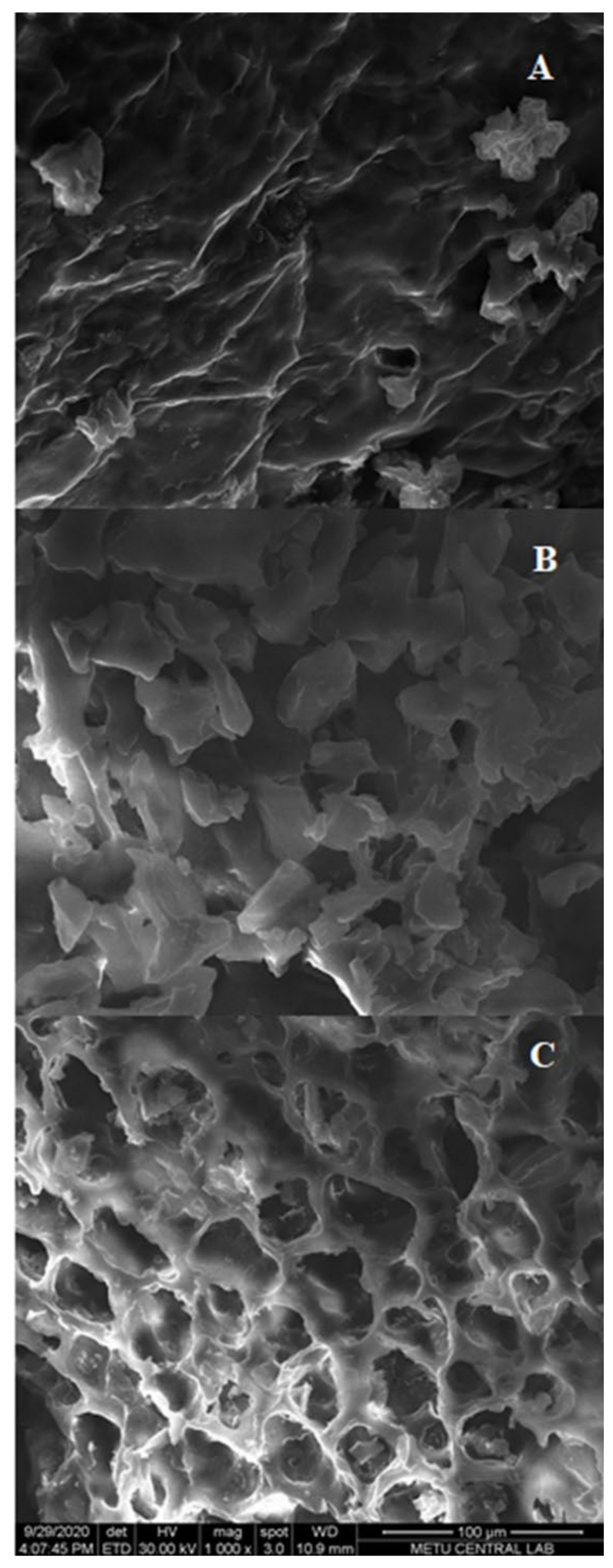
SEM results (**A**) CSE (**B**) HHPE (500 MPa for 15 min) (**C**) UAE (60% Amplitude for 15 min).

The morphological changes of different extraction techniques were characterized by scanning electron microscopy (SEM) analysis and the results are depicted in Figure 6. After CSE and HHPE, no cell damage was observed (Figure 6A,B). However, more contact area was detected and this might increase the phenolic transfer rate of HHPE. A similar result was also reported by our research group for the extraction of sour cherry pomace by different extraction methods [26]. For UAE, cell damage was observed compared to CSE and HHPE (Figure 6C). This result has also been shown in the literature [26,41,42]. At UAE treatment, acoustic cavitation occurs and this leads to micro fissures and microchannels on the matrix surface so UAE increases the transfer of phenolic compounds into the solvent [43,44]. In brief, SEM analysis indicated that the eco-friendly techniques caused an increase in the release of phenolics comparing to CSE basically due to the morphological changes.

## 3. Materials and Methods

### 3.1. Materials

Spent coffee grounds (SCG, coffee Arabica) was provided from a local branch of a national coffee chain located at Middle East Technical University Campus in Ankara, Turkey (September 2020) and stored at −18 °C until usage. The initial moisture content of the SCG was 22.8 ± 0.5%.

### 3.2. Conventional Solvent Extraction (CSE)

The conventional solvent extraction (CSE) technique was applied according to Altemimi et al. [45] with some modifications. In brief, a SCG-% 80 methanol solution (10% *w*/*v*) was prepared. Then, the mixture was placed in a water bath (WiseCircu, Seoul, Korea) at 50 °C for 30 min to solubilize phenolic compounds from SCG. Then, the mixture was filtered by filter paper (Whatman No.1), and the extract was stored at 4 °C until further analysis. CSE technique was indicated as control when presenting the results.

### 3.3. High Hydrostatic Pressure-Assisted Extraction (HHPE)

High Hydrostatic Pressure-Assisted Extraction (HHPE) treatment was applied by using 760.0118 type pressure equipment (SITEC-Sieber Engineering AG, Zurich, Switzerland). The vessel volume was 100 mL with diameter 24 mm and length 153 mm. A built-in heating–cooling system (Huber Circulation Thermostat, Offenburg, Germany) was used to control treatment temperature measured by thermocouple. The equipment consists of a pressurization chamber, two end closures, a means for restraining the end closures, a pressure pump, a hydraulic unit, and a temperature control device. A mixture of water and glycol was used as pressure-transmitting medium and it was heated prior to pressurization to reach the treatment temperature. The pressure release time was less than 20 s for each so the pressurization time reported in this study did not contain the pressure increase and release times. Prepared SCG-% 80 methanol solutions (10% *w*/*v*) were pressurized in 25 mL sterile polyethylene cryotubes (LP Italiana SPA, Milano, Italy) at 300, 400, and 500 MPa for 5, 10, and 15 min at constant temperature as 25 °C. After HHPE treatment, filtration of the mixture was performed by filter paper (Whatman No.1), and the extract was stored at 4 °C till further analysis.

### 3.4. Ultrasound-Assisted Extraction (UAE)

Ultrasound-Assisted Extraction (UAE) treatment was applied with a Heilscher UP400S system (Dr.Heilscher GmbH, Teltow, Germany) at 24 kHz, 400 W. At all UAE treatments, a titanium alloy sonotrode (H3, Dr. Heilscher GmbH) with ID 3 mm was used. To keep constant temperature of the mixture for all UAE treatments, ice bath was used to keep the constant temperature of the mixture at roughly 25 ± 1 °C. Prepared SCG-% 80 methanol solutions (10% *w*/*v*) were treated at 25 kHz with different amplitudes (40, 50, and 60%) at different treatment times (5, 10, and 15 min). The probe was put into the solution from the center in such a way that the height was 1/3 of the sample height from the bottom. After treatment, the mixture was filtered by using filter paper (Whatman No.1) and the extract was stored at 4 °C till further analysis.

### 3.5. Total Phenolic Content (TPC)

The Folin-Ciocalteu assay was performed to calculate the TPC of samples according to Okur et al. [26]. In brief, 0.75 mL Folin-Ciocalteu solution (10% *v*/*v*) was added to 100 µL extract. Next, the mixture was kept for 5 min at room temperature, and 0.75 mL of sodium carbonate solution (7.5 g/L) was added. Then, the mixture was kept for 1 h in the dark. Finally, the absorbance of the samples was measured at 725 nm by using spectrophotometer (Shimadzu UV-1700, Tokyo, Japan). The gallic acid calibration curve was used as a standard to quantify the TPC value of samples, and TPC results were expressed as mg gallic acid equivalent (GAE)/100 g fresh weight (FW).

### 3.6. Antioxidant Activity (AA)

#### 3.6.1. DPPH Assay

An aliquot of 100 µL extract was mixed with 3.9 mL of 0.1 mM prepared 1, 1-diphenyl-2-picrylhydrazyl (DPPH) solution prepared with 80% methanol solution and the mixture was stored in dark at room temperature for 30 min. Then, the absorbance of the mixture was measured at 517 nm (Shimadzu UV-1700). Antioxidant activity results were given as % inhibition of DPPH activity and the results were calculated according to the following formula [26]:

%inhibition of DPPH activity = [1 − (As/Ac)] × 100(1)

where As is the absorbance value of the sample, Ac is the absorbance value of control.

#### 3.6.2. Ferric Reducing Antioxidant Power (FRAP)

The total antioxidant activity (AA) was also performed by using the ferric reducing ability of plasma FRAP assay by Benzie and Strain, [46]. The FRAP assay utilizes antioxidants as reductants in a redox-linked colorimetric method. At low pH, the reduction of ferric tripyridyl triazine (Fe III TPTZ) complex to ferrous form was detected by measuring the change in absorption at 593 nm. Three ml of FRAP reagent [(a) acetate buffer (300 mM pH 3.6) was prepared by weighing 3.1 g sodium acetate trihydrate (CH_3_COONa · 3H_2_O) and adding 16 mL of glacial acetic acid to make the volume to 1 L with distilled water. (b) TPTZ (2, 4, 6-tripyridyl-s- triazine) (MW 312.34) 10 mM in 40 mM HCl (MW 36.46) (c) FeCl_3_ · 6H_2_O (MW 270.30) 20 mM. The FRAP reagent was prepared by mixture of a, b and c with the ratio of 10:1:1 respectively and mixed with 100 μL sample and absorbance at 593 nm was measured at t = 0 min after thorough vortexing. Then, samples were put in water bath (WiseCircu) at 37 °C and absorbance of samples were again measured after 4 min. The standard ferrous sulfate solution (FeSO_4_) curve was used as a standard to quantify the antioxidant activity of samples, and the FRAP results were calculated as mmol FeSO_4_/100 g fresh weight (FW).

### 3.7. Scanning Electron Microscopy (SEM)

SEM analysis was performed to find the morphological modifications after different extraction techniques, by using electron microscope (Nova NanoSEM 430, FEI, OR, USA). Before imaging, the samples were lyophilized for two days (Zhejiang ValueMechanical & Electrical Products Co. Ltd., Wenling City, China) and coated with a thin layer of Au–Pd at room temperature.

### 3.8. Infrared Spectroscopy (IR)

Infrared (IR) spectra were recorded using a FTIR spectrometer (Shimadzu Corporation) to assess the changes in chemical structures of phenolic compounds caused by extraction techniques. The lyophilized sample was placed over the attenuated total refection (ATR) crystal and the FTIR spectra was measured in the 4000 cm^−1^ to 600 cm^−1^ range by the addition 32 scans with a resolution of 2 cm^−1^.

### 3.9. High-Performance Liquid Chromatography (HPLC)

High-performance liquid chromatography (HPLC, Shimadzu Corporation) was performed in different treated samples to quantify caffeic acid and chlorogenic acid. HPLC system included an autosampler (SIL-10ADvp), a quaternary pump (LC-10ADvp) and a diode array detector (DAD). All the samples were passed through 0.45 μm nylon filter membranes. A Simultaneous determination of chlorogenic acid and caffeic acid, was performed in a Shimadzu HPLC. An Eclipse XDB-C18 column (Agilent197, Palo Alto, CA, USA) (250 × 4.60 mm) with a particle size of 5 µm was used. The mobile phase included 3% acetic acid in water (A) and methanol (B). Extracts were eluted based on following steps: the gradient was started with 7% B to reach 28% B at 20 min, 25% B at 28 min, 30% B at 35 min, 30% B at 50 min, 33% B at 60 min, 42% B at 62 min, 50% B at 70 min, 70% B at 73 min, 80% B at 75 min, 100% B at 80 min and 7% B at 81 min. The column temperature and flow rate were 30 °C and 0.8 mL/min respectively. The phenolics were found and quantified at 278 nm [47]. For the quantification of SCG, the external standards were used. The good linearity (correlation coefficient values (R^2^ > 0.999) was achieved in a relatively wide concentration ranging from 0 to 2 ppm for chlorogenic acid and caffeic acid. Regression equation, R^2^ values, the limit of detection (LOD) and quantification (LOQ) values were summarized in Table 2.

### 3.10. Statistical Analysis

SigmaPlot (Ver.14, Systat Software Inc., San Jose, CA, USA) was used for data analysis. Analysis of Variance (ANOVA) was carried out to detect the differences between samples. Furthermore, Tukey’s multiple range test was used to interpret significant differences between the experimental mean values (*p* < 0.05).

## 4. Conclusions

To the best of our knowledge, this is the first study reporting the effect of environmentally-friendly extraction techniques on the recovery of phenolic compounds from waste spent coffee grounds (SCG). Both eco-friendly extraction methods (HHPE and UAE) increased the TPC and AA significantly (*p* ≤ 0.05). Also, IR results indicated that there was no significant difference in the chemical structures of the phenolic compounds recovered using either UAE or HHPE as compared to those recovered by CSE. The morphological changes taking place during these methods led to an increase in the mass transfer of phenolic substances. According to HPLC results, HHPE and UAE increased the chlorogenic and caffeic acid content. Among these, UAE produced more chlorogenic and caffeic acid than HHPE as supported by TPC and AA results. In brief, our research shows that HHPE and UAE are not only suitable and convenient but also environmentally-friendly and fast extraction methods as compared to CSE for improvıng the recovery of phenolic compounds from SCG waste.

## Figures and Tables

**Table 1:** Individual phenolic compounds from SCG extracted by CSE, HHPE (500 MPa for 15 min) and UAE (60% Amplitude for 15 min).

	Chlorogenic Acid	Caffeic Acid
CSE	24.0 ± 0.3 ^c^	2.2 ± 0.1 ^c^
HHPE	81.2 ± 1.1 ^b^	5.4 ± 0.5 ^a,b^
UAE	85.0 ± 0.6 ^a^	6.1 ± 0.2 ^a^

^a–c^ Different small letters show significant differences (*p* ≤ 0.05).

**Table 2:** Calibration data for chlorogenic acid and caffeic acid.

Compound	Regression Equation (y = ax + b)	R^2^	LOD (ppm)	LOQ (ppm)
Chlorogenic acid	−1568.37x + 31,041.23	0.99994	0.02	0.07
Caffeic Acid	600.75x + 76,114.15	0.99948	0.05	0.14

## Sample Availability

Samples of the compounds are not available from the authors.
